# 4-Bromo-2-(5-bromo­thio­phen-2-yl)-1-[(5-bromo­thio­phen-2-yl)meth­yl]-5,6-dimethyl-1*H*-benzimidazole

**DOI:** 10.1107/S160053681400628X

**Published:** 2014-03-26

**Authors:** H. Cristina Geiger, James S. Donohoe, David K. Geiger

**Affiliations:** a Department of Chemistry, State University of New York-College at Geneseo, 1 College Circle, Geneseo, NY 14454, USA

## Abstract

The title compound, C_18_H_13_Br_3_N_2_S_2_, was obtained *via* the reaction of *N*-bromo­succinamide with 5,6-dimethyl-2-(thio­phen-2-yl)-1-[(thio­phen-2-yl)meth­yl]-1*H*-benzimidazole. The compound exhibits rotational disorder of the 5-bromo­thio­phen-2-yl substituent with a refined major:minor occupancy ratio of 0.876 (7):0.124 (7). The 5-bromo­thio­phen-2-yl mean plane is canted to the benzimidazole plane by 20.0 (4) and 21 (4)° in the major and minor components, respectively. In the crystal, weak C—H⋯N inter­actions link the mol­ecules into infinite *C*(7) chains along the 2_1_ axes.

## Related literature   

Bromination of thio­phenes using *N*-bromo­succinamide has been reported by Arsenyan *et al.* (2010 ▶). For the structure of 5,6-di­methyl­benzimidazole, see: Lee & Scheidt (1986 ▶). For the structure of 2-(thio­phen-2-yl)-1-(thio­phen-2-ylmeth­yl)-1*H*-benzimidazole, see: Geiger *et al.* (2012 ▶). For the 5-chloro derivative, see: Geiger & Nellist (2013*a* ▶), for the 6-chloro derivative, see: Geiger & Nellist (2013*b* ▶) and for the 6-bromo derivative, see: Geiger & Destefano (2012 ▶). For a discussion of the biological activity of benzimidazole derivatives, see: López-Rodríguez *et al.* (1999 ▶); Horton *et al.* (2003 ▶).
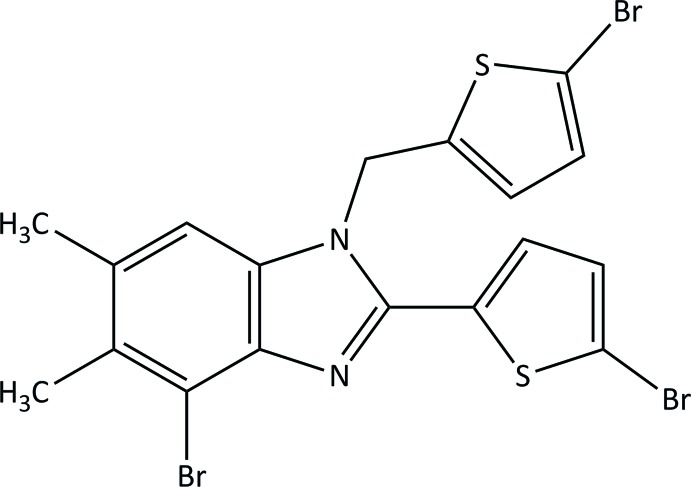


## Experimental   

### 

#### Crystal data  

C_18_H_13_Br_3_N_2_S_2_
*M* *_r_* = 561.15
Monoclinic, 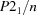

*a* = 13.6796 (13) Å
*b* = 9.6144 (8) Å
*c* = 14.8093 (15) Å
β = 98.305 (3)°
*V* = 1927.3 (3) Å^3^
*Z* = 4
Mo *K*α radiation
μ = 6.50 mm^−1^
*T* = 200 K
0.60 × 0.40 × 0.10 mm

#### Data collection  

Bruker SMART X2S benchtop diffractometer
Absorption correction: multi-scan (*SADABS*; Bruker, 2013 ▶) *T*_min_ = 0.35, *T*_max_ = 0.56
12301 measured reflections
3561 independent reflections
2810 reflections with *I* > 2σ(*I*)
*R*_int_ = 0.036

#### Refinement  

*R*[*F* ^2^ > 2σ(*F* ^2^)] = 0.056
*wR*(*F* ^2^) = 0.179
*S* = 1.06
3561 reflections
247 parameters
16 restraints
H-atom parameters constrained
Δρ_max_ = 0.87 e Å^−3^
Δρ_min_ = −1.90 e Å^−3^

### 

Data collection: *APEX2* (Bruker, 2013 ▶); cell refinement: *SAINT* (Bruker, 2013 ▶); data reduction: *SAINT*; program(s) used to solve structure: *SHELXS97* (Sheldrick, 2008 ▶); program(s) used to refine structure: *SHELXL2013* (Sheldrick, 2013 ▶); molecular graphics: *PLATON* (Spek, 2009 ▶) and *Mercury* (Macrae *et al.*, 2008 ▶); software used to prepare material for publication: *publCIF* (Westrip, 2010 ▶).

## Supplementary Material

Crystal structure: contains datablock(s) global, I. DOI: 10.1107/S160053681400628X/rz5112sup1.cif

Structure factors: contains datablock(s) I. DOI: 10.1107/S160053681400628X/rz5112Isup2.hkl

Supporting information file. DOI: 10.1107/S160053681400628X/rz5112Isup3.mol

Supporting information file. DOI: 10.1107/S160053681400628X/rz5112Isup4.cml

CCDC reference: 992876

Additional supporting information:  crystallographic information; 3D view; checkCIF report

## Figures and Tables

**Table 1:** Hydrogen-bond geometry (Å, °)

*D*—H⋯*A*	*D*—H	H⋯*A*	*D*⋯*A*	*D*—H⋯*A*
C14—H14⋯N2^i^	0.95	2.57	3.461 (10)	156

Symmetry code: (i) 
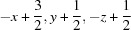
.

**Fig. 1.**
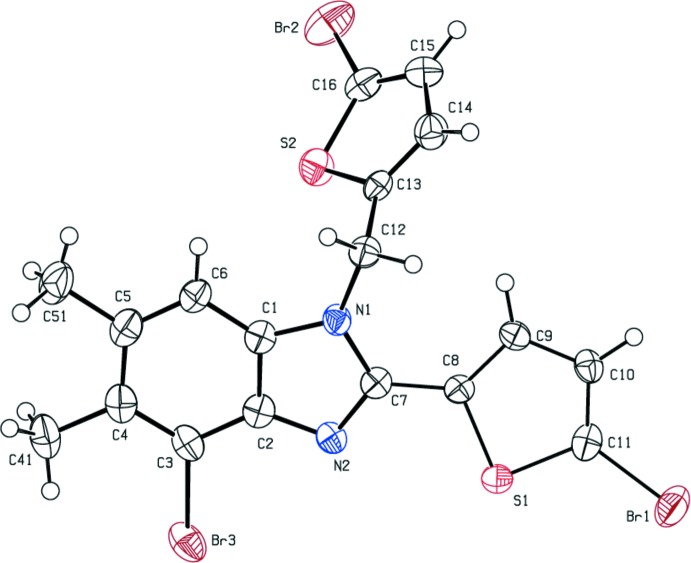
The molecular structure of the title compound with anisotropic displacement parameters of nonhydrogen atoms drawn at the 50% probability level. Only the major contributor to the disordered bromothiophene substituent is shown.

**Fig. 2.**
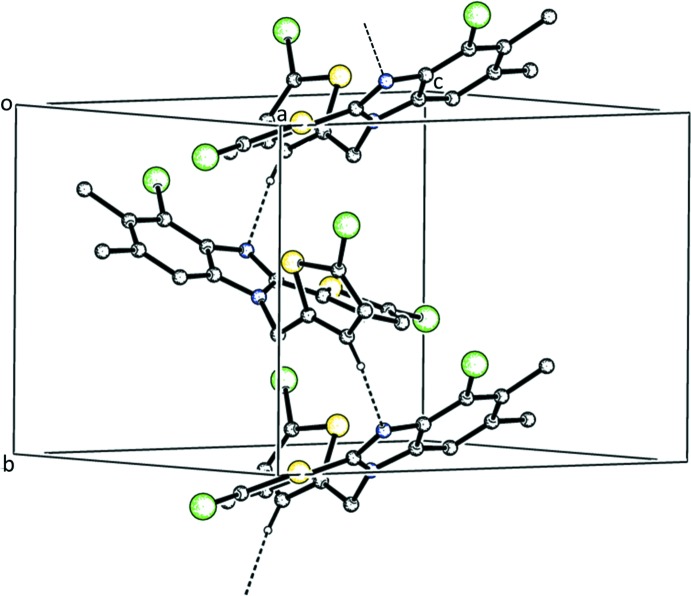
A packing diagram emphasizing the weak C—H···N hydrogen bonds that link molecules along the 21 screw axes. All hydrogen atoms except H14 have been omitted for clarity.

## supplementary crystallographic information

### 1. Comment

Benzimidazole derivatives have numerous pharmacological uses. Examples include inhibitors of serotonin activated neurotransmission drugs (López-Rodríguez *et al.*, 1999) and antiarrhythmic, antihistamine, antiulcer, anticancer, fungicidal, and anthelmintical drugs (Horton *et al.*, 2003). The title compound was prepared as part of our efforts to characterize benzimidazole analogues with thiophene substitutents (Geiger & Nellist, 2013*a*; Geiger & Nellist, 2013*b*; Geiger & Destefano, 2012; Geiger *et al.*, 2012).

The title compound was prepared serendipitously during the attempted bromination of the methyl substituents of 5,6-dimethyl-2-(thiophen-2-yl)-1-(thiophen-2-ylmethyl)-1*H*- benzimidazole. Bromination of the positions adjacent to the sulfur atom in thiophene and thiophene derivatives with *N*-bromosuccinamide (NBS)using ultrasonic irradiation has been reported by Arsenyan *et al.* (2010). In all cases, bromination occurs at sites adjacent to the sulfur atom. Based on this observation, it is not surprising that the two thiophene groups are brominated in the title complex. However, bromination of the benzene ring using NBS is not expected, as free radical bromination is expected to occur at the benzylic positions.

Compound I crystallizes with one molecule in the asymmetric unit. A perspective view of the molecule with the atom-labeling scheme is shown in figure 1. The benzimidazole ring system is essentially planar. The largest deviation from planarity is 0.017 (6) Å for C5. The 2-(5-bromothiophen-2-yl) plane is canted 20.0 (4)*^o^* and 21 (4)*^o^* to the benzimidazole plane in the major and minor disorder components, respectively.

The extended structure exhibits chains along the 2_1_ screw axes formed by weak intermolecular C—H···N hydrogen bonds (Table 1) involving one of the 5-bromothiophen-2-ylmethyl hydrogen atoms (H14) and the unsubstitued benzimidazole nitrogen atom (N2). The result is infinite *C*(7) chains. Figure 2 displays a packing diagram exhibiting the chains parallel to [0 1 0].

An additional close contact between bromine atoms on molecules related by the glide plane is observed. The Br2···Br10 distance is 3.041 (19) Å (Br10 is part of the minor component of the disordered 5-bromothiophen-2-yl substituent.) The Br2···Br1 (Br1 is the bromine of the major component) distance is 3.653 (3) Å.

### 2. Experimental

The title compound was prepared by a photoinitiated reaction of N-bromosuccimide (1.10 g, 6.18 mmole) and 5,6-dimethyl-2-(thiophen-2-yl)-1-(thiophen-2-ylmethyl)-1*H*-benzimidazole (0.864 g, 2.66 mmole) in refluxing carbon tetrachloride (20 mL). Based on GC—MS results, the material isolated was a mixture of a mono-, di- and tri-brominated components. Attempts to separate the components by column chromatography or recrystallization were unsuccessful. Based on a comparison of a ^1^H NMR spectrum of the product mixture and simulated spectra of mono-, di- and tri-brominated products, the reaction product mixture is approximately 50% tri-brominated species. Single crystals of the tribromo component (the title compound) were obtained by slow vapor diffusion of hexanes into a chloroform solution of the product mixture.

### 3. Refinement

During the later stages of refinement, it became obvious that the molecule exhibited rotational disorder about the 5-bromothiophen-2-yl substituent. The disorder was successfully modeled using the metrics of the major component to define the minor component. Similarity restraints were used for the bond distances using SAME and anisotropic displacement parameters of the minor component atoms were constrained to those of the major component using EADP. The structure converged with a refined major:minor occupancy ratio of 0.876 (7):0.124 (7). All hydrogen atoms were observed in difference Fourier fourier maps. The H atoms were refined using a riding model with a C—H distance of 0.99 Å for the methylene carbon atoms, 0.98 Å for the methyl carbon atoms and 0.95 Å for the phenyl and thiophene carbon atoms. The methyl C—H hydrogen atom isotropic displacement parameters were set using the approximation *U*_iso_(H) = 1.5*U*_eq_(C). All other C—H hydrogen atom isotropic displacement parameters were set using the approximation *U*_iso_(H) = 1.2*U*_eq_(C).

### Crystal data

**Table:**

C_18_H_13_Br_3_N_2_S_2_	*F*(000) = 1088
*M**_r_* = 561.15	*D*_x_ = 1.934 Mg m^−^^3^
Monoclinic, *P*2_1_/*n*	Mo *K*α radiation, λ = 0.71073 Å
Hall symbol: -P 2yn	Cell parameters from 4288 reflections
*a* = 13.6796 (13) Å	θ = 2.5–25.0°
*b* = 9.6144 (8) Å	µ = 6.50 mm^−^^1^
*c* = 14.8093 (15) Å	*T* = 200 K
β = 98.305 (3)°	Plate, clear colourless
*V* = 1927.3 (3) Å^3^	0.60 × 0.40 × 0.10 mm
*Z* = 4	

### Data collection

**Table:**

Bruker SMART X2S benchtop diffractometer	3561 independent reflections
Radiation source: sealed microfocus tube	2810 reflections with *I* > 2σ(*I*)
Doubly curved silicon crystal monochromator	*R*_int_ = 0.036
Detector resolution: 8.3330 pixels mm^-1^	θ_max_ = 25.5°, θ_min_ = 1.9°
ω scans	*h* = −16→16
Absorption correction: multi-scan (*SADABS*; Bruker, 2013)	*k* = −11→11
*T*_min_ = 0.35, *T*_max_ = 0.56	*l* = −17→11
12301 measured reflections	

### Refinement

**Table:**

Refinement on *F*^2^	Primary atom site location: structure-invariant direct methods
Least-squares matrix: full	Secondary atom site location: difference Fourier map
*R*[*F*^2^ > 2σ(*F*^2^)] = 0.056	Hydrogen site location: inferred from neighbouring sites
*wR*(*F*^2^) = 0.179	H-atom parameters constrained
*S* = 1.06	*w* = 1/[σ^2^(*F*_o_^2^) + (0.0876*P*)^2^ + 13.3538*P*] where *P* = (*F*_o_^2^ + 2*F*_c_^2^)/3
3561 reflections	(Δ/σ)_max_ < 0.001
247 parameters	Δρ_max_ = 0.87 e Å^−^^3^
16 restraints	Δρ_min_ = −1.90 e Å^−^^3^

### Special details

**Table:**

Geometry. All e.s.d.'s (except the e.s.d. in the dihedral angle between two l.s. planes) are estimated using the full covariance matrix. The cell e.s.d.'s are taken into account individually in the estimation of e.s.d.'s in distances, angles and torsion angles; correlations between e.s.d.'s in cell parameters are only used when they are defined by crystal symmetry. An approximate (isotropic) treatment of cell e.s.d.'s is used for estimating e.s.d.'s involving l.s. planes.
Refinement. Refinement of *F*^2^ against ALL reflections. The weighted *R*-factor *wR* and goodness of fit *S* are based on *F*^2^, conventional *R*-factors *R* are based on *F*, with *F* set to zero for negative *F*^2^. The threshold expression of *F*^2^ > σ(*F*^2^) is used only for calculating *R*-factors(gt) *etc*. and is not relevant to the choice of reflections for refinement. *R*-factors based on *F*^2^ are statistically about twice as large as those based on *F*, and *R*- factors based on ALL data will be even larger.

### Fractional atomic coordinates and isotropic or equivalent isotropic displacement parameters (Å^2^)

**Table:**

	*x*	*y*	*z*	*U*_iso_*/*U*_eq_	Occ. (<1)
C1	0.6567 (5)	0.4489 (7)	0.0616 (5)	0.0297 (15)	
C2	0.5806 (5)	0.3747 (7)	0.0935 (5)	0.0291 (15)	
C3	0.5164 (5)	0.2979 (7)	0.0299 (5)	0.0328 (16)	
C4	0.5271 (6)	0.2972 (7)	−0.0623 (5)	0.0345 (17)	
C5	0.6069 (6)	0.3752 (9)	−0.0909 (5)	0.0388 (18)	
C6	0.6698 (6)	0.4519 (8)	−0.0296 (5)	0.0341 (16)	
H6	0.7211	0.5058	−0.0491	0.041*	
C41	0.4614 (7)	0.2091 (9)	−0.1300 (6)	0.048 (2)	
H41A	0.4983	0.1275	−0.146	0.071*	
H41B	0.4395	0.2637	−0.185	0.071*	
H41C	0.4037	0.1787	−0.1029	0.071*	
C51	0.6220 (7)	0.3744 (12)	−0.1887 (5)	0.055 (2)	
H51A	0.6732	0.442	−0.1979	0.083*	
H51B	0.56	0.3992	−0.2271	0.083*	
H51C	0.6426	0.2813	−0.2053	0.083*	
Br3	0.41595 (6)	0.19379 (9)	0.07516 (6)	0.0461 (3)	
N1	0.7086 (4)	0.5125 (6)	0.1385 (4)	0.0291 (13)	
C7	0.6621 (5)	0.4710 (7)	0.2112 (5)	0.0282 (15)	
N2	0.5860 (4)	0.3888 (6)	0.1866 (4)	0.0284 (13)	
C8	0.6925 (12)	0.5165 (15)	0.3059 (6)	0.0252 (18)	0.876 (7)
C9	0.7801 (11)	0.5660 (14)	0.3497 (7)	0.031 (2)	0.876 (7)
H9	0.8365	0.5803	0.3203	0.037*	0.876 (7)
C10	0.7787 (8)	0.5937 (9)	0.4431 (6)	0.030 (2)	0.876 (7)
H10	0.8334	0.6288	0.4835	0.036*	0.876 (7)
C11	0.6903 (8)	0.5643 (9)	0.4674 (5)	0.032 (2)	0.876 (7)
S1	0.6069 (8)	0.5018 (9)	0.3795 (3)	0.0336 (12)	0.876 (7)
Br1	0.65371 (11)	0.5955 (3)	0.58337 (8)	0.0544 (5)	0.876 (7)
C80	0.685 (8)	0.502 (10)	0.302 (3)	0.0252 (18)	0.124 (7)
C90	0.775 (7)	0.543 (11)	0.347 (4)	0.031 (2)	0.124 (7)
H90	0.8304	0.5603	0.3166	0.037*	0.124 (7)
C100	0.777 (5)	0.557 (9)	0.442 (4)	0.030 (2)	0.124 (7)
H100	0.8338	0.5836	0.4829	0.036*	0.124 (7)
C110	0.689 (4)	0.529 (7)	0.4662 (19)	0.032 (2)	0.124 (7)
S10	0.602 (6)	0.480 (8)	0.377 (2)	0.0336 (12)	0.124 (7)
Br10	0.6580 (9)	0.530 (2)	0.5862 (6)	0.0544 (5)	0.124 (7)
C12	0.7869 (5)	0.6148 (7)	0.1354 (5)	0.0312 (16)	
H12A	0.7851	0.6824	0.1856	0.037*	
H12B	0.7734	0.6666	0.0772	0.037*	
C13	0.8884 (5)	0.5541 (7)	0.1435 (4)	0.0267 (15)	
C14	0.9740 (6)	0.6115 (9)	0.1828 (5)	0.0399 (18)	
H14	0.9773	0.6971	0.2151	0.048*	
C15	1.0593 (6)	0.5319 (9)	0.1713 (6)	0.0426 (19)	
H15	1.1251	0.5573	0.195	0.051*	
C16	1.0341 (6)	0.4152 (8)	0.1220 (5)	0.0387 (18)	
S2	0.90879 (16)	0.3984 (2)	0.09103 (14)	0.0396 (5)	
Br2	1.11809 (9)	0.28087 (11)	0.08705 (7)	0.0637 (4)	

### Atomic displacement parameters (Å^2^)

**Table:**

	*U*^11^	*U*^22^	*U*^33^	*U*^12^	*U*^13^	*U*^23^
C1	0.036 (4)	0.020 (3)	0.032 (4)	0.003 (3)	0.005 (3)	0.001 (3)
C2	0.034 (4)	0.021 (3)	0.032 (4)	0.009 (3)	0.003 (3)	0.003 (3)
C3	0.033 (4)	0.025 (4)	0.037 (4)	0.003 (3)	−0.005 (3)	0.003 (3)
C4	0.037 (4)	0.029 (4)	0.035 (4)	0.015 (3)	−0.004 (3)	−0.001 (3)
C5	0.040 (4)	0.046 (5)	0.029 (4)	0.019 (4)	0.002 (3)	0.004 (3)
C6	0.038 (4)	0.035 (4)	0.029 (4)	0.005 (3)	0.007 (3)	0.002 (3)
C41	0.049 (5)	0.050 (5)	0.039 (4)	0.007 (4)	−0.010 (4)	−0.009 (4)
C51	0.049 (5)	0.086 (7)	0.030 (4)	0.023 (5)	0.000 (4)	0.001 (4)
Br3	0.0447 (5)	0.0381 (5)	0.0518 (5)	−0.0119 (4)	−0.0055 (4)	0.0037 (4)
N1	0.034 (3)	0.026 (3)	0.027 (3)	−0.006 (3)	0.004 (2)	−0.002 (2)
C7	0.033 (4)	0.021 (3)	0.031 (4)	0.002 (3)	0.006 (3)	0.001 (3)
N2	0.031 (3)	0.021 (3)	0.032 (3)	−0.002 (2)	0.002 (2)	0.001 (2)
C8	0.030 (5)	0.017 (4)	0.029 (3)	−0.003 (3)	0.007 (3)	0.002 (3)
C9	0.030 (4)	0.029 (6)	0.033 (4)	−0.007 (4)	0.005 (3)	−0.001 (3)
C10	0.036 (4)	0.023 (6)	0.029 (4)	−0.006 (4)	0.000 (3)	0.001 (4)
C11	0.050 (5)	0.019 (5)	0.027 (4)	−0.002 (4)	0.006 (3)	0.003 (3)
S1	0.0328 (17)	0.038 (4)	0.0307 (10)	−0.0099 (19)	0.0077 (9)	−0.0001 (11)
Br1	0.0781 (7)	0.0542 (15)	0.0354 (5)	−0.0216 (8)	0.0230 (5)	−0.0079 (6)
C80	0.030 (5)	0.017 (4)	0.029 (3)	−0.003 (3)	0.007 (3)	0.002 (3)
C90	0.030 (4)	0.029 (6)	0.033 (4)	−0.007 (4)	0.005 (3)	−0.001 (3)
C100	0.036 (4)	0.023 (6)	0.029 (4)	−0.006 (4)	0.000 (3)	0.001 (4)
C110	0.050 (5)	0.019 (5)	0.027 (4)	−0.002 (4)	0.006 (3)	0.003 (3)
S10	0.0328 (17)	0.038 (4)	0.0307 (10)	−0.0099 (19)	0.0077 (9)	−0.0001 (11)
Br10	0.0781 (7)	0.0542 (15)	0.0354 (5)	−0.0216 (8)	0.0230 (5)	−0.0079 (6)
C12	0.039 (4)	0.025 (3)	0.031 (4)	−0.006 (3)	0.008 (3)	0.003 (3)
C13	0.035 (4)	0.023 (3)	0.024 (3)	0.001 (3)	0.009 (3)	0.001 (3)
C14	0.044 (5)	0.037 (4)	0.039 (4)	0.000 (4)	0.007 (4)	−0.006 (3)
C15	0.032 (4)	0.052 (5)	0.046 (5)	0.000 (4)	0.011 (3)	−0.002 (4)
C16	0.041 (5)	0.042 (4)	0.036 (4)	0.009 (4)	0.014 (3)	0.005 (3)
S2	0.0445 (11)	0.0302 (10)	0.0444 (11)	0.0010 (9)	0.0071 (9)	−0.0083 (8)
Br2	0.0795 (7)	0.0563 (6)	0.0619 (6)	0.0217 (5)	0.0329 (5)	0.0114 (5)

### Geometric parameters (Å, º)

**Table:**

C1—C6	1.389 (10)	C9—C10	1.410 (10)
C1—N1	1.393 (9)	C9—H9	0.95
C1—C2	1.399 (10)	C10—C11	1.341 (11)
C2—N2	1.376 (9)	C10—H10	0.95
C2—C3	1.402 (10)	C11—S1	1.711 (8)
C3—C4	1.394 (11)	C11—Br1	1.882 (8)
C3—Br3	1.899 (8)	C80—C90	1.36 (2)
C4—C5	1.439 (12)	C80—S10	1.717 (19)
C4—C41	1.506 (11)	C90—C100	1.41 (2)
C5—C6	1.372 (11)	C90—H90	0.95
C5—C51	1.492 (11)	C100—C110	1.34 (2)
C6—H6	0.95	C100—H100	0.95
C41—H41A	0.98	C110—S10	1.710 (19)
C41—H41B	0.98	C110—Br10	1.884 (18)
C41—H41C	0.98	C12—C13	1.494 (10)
C51—H51A	0.98	C12—H12A	0.99
C51—H51B	0.98	C12—H12B	0.99
C51—H51C	0.98	C13—C14	1.349 (11)
N1—C7	1.387 (9)	C13—S2	1.728 (7)
N1—C12	1.460 (9)	C14—C15	1.426 (11)
C7—N2	1.317 (9)	C14—H14	0.95
C7—C80	1.37 (5)	C15—C16	1.356 (12)
C7—C8	1.470 (11)	C15—H15	0.95
C8—C9	1.363 (10)	C16—S2	1.717 (8)
C8—S1	1.717 (7)	C16—Br2	1.852 (8)
			
C6—C1—N1	131.5 (7)	C8—C9—C10	113.4 (7)
C6—C1—C2	123.1 (7)	C8—C9—H9	123.3
N1—C1—C2	105.3 (6)	C10—C9—H9	123.3
N2—C2—C1	110.7 (6)	C11—C10—C9	111.3 (7)
N2—C2—C3	131.5 (7)	C11—C10—H10	124.4
C1—C2—C3	117.8 (7)	C9—C10—H10	124.4
C4—C3—C2	121.1 (7)	C10—C11—S1	113.5 (6)
C4—C3—Br3	121.7 (6)	C10—C11—Br1	125.5 (6)
C2—C3—Br3	117.1 (5)	S1—C11—Br1	120.9 (5)
C3—C4—C5	118.5 (7)	C11—S1—C8	90.7 (4)
C3—C4—C41	121.2 (8)	C90—C80—C7	127 (6)
C5—C4—C41	120.2 (7)	C90—C80—S10	111.0 (17)
C6—C5—C4	121.0 (7)	C7—C80—S10	122 (6)
C6—C5—C51	118.9 (8)	C80—C90—C100	114 (2)
C4—C5—C51	120.0 (8)	C80—C90—H90	123.2
C5—C6—C1	118.4 (7)	C100—C90—H90	123.2
C5—C6—H6	120.8	C110—C100—C90	111 (2)
C1—C6—H6	120.8	C110—C100—H100	124.5
C4—C41—H41A	109.5	C90—C100—H100	124.5
C4—C41—H41B	109.5	C100—C110—S10	113.7 (16)
H41A—C41—H41B	109.5	C100—C110—Br10	126 (2)
C4—C41—H41C	109.5	S10—C110—Br10	120 (2)
H41A—C41—H41C	109.5	C110—S10—C80	90.7 (13)
H41B—C41—H41C	109.5	N1—C12—C13	114.3 (6)
C5—C51—H51A	109.5	N1—C12—H12A	108.7
C5—C51—H51B	109.5	C13—C12—H12A	108.7
H51A—C51—H51B	109.5	N1—C12—H12B	108.7
C5—C51—H51C	109.5	C13—C12—H12B	108.7
H51A—C51—H51C	109.5	H12A—C12—H12B	107.6
H51B—C51—H51C	109.5	C14—C13—C12	127.9 (7)
C7—N1—C1	105.8 (6)	C14—C13—S2	111.2 (6)
C7—N1—C12	129.7 (6)	C12—C13—S2	120.7 (5)
C1—N1—C12	124.1 (6)	C13—C14—C15	113.8 (7)
N2—C7—C80	118 (4)	C13—C14—H14	123.1
N2—C7—N1	113.0 (6)	C15—C14—H14	123.1
C80—C7—N1	129 (4)	C16—C15—C14	111.1 (7)
N2—C7—C8	123.1 (7)	C16—C15—H15	124.5
N1—C7—C8	123.9 (7)	C14—C15—H15	124.5
C7—N2—C2	105.2 (6)	C15—C16—S2	112.9 (6)
C9—C8—C7	131.8 (10)	C15—C16—Br2	127.4 (6)
C9—C8—S1	111.1 (6)	S2—C16—Br2	119.7 (5)
C7—C8—S1	117.1 (8)	C16—S2—C13	91.1 (4)

### Hydrogen-bond geometry (Å, º)

**Table:**

*D*—H···*A*	*D*—H	H···*A*	*D*···*A*	*D*—H···*A*
C14—H14···N2^i^	0.95	2.57	3.461 (10)	156

Symmetry code: (i) −*x*+3/2, *y*+1/2, −*z*+1/2.
